# A Prognostic Score for Patients with Intermediate-Stage Hepatocellular Carcinoma Treated with Transarterial Chemoembolization

**DOI:** 10.1371/journal.pone.0125244

**Published:** 2015-04-28

**Authors:** Sadahisa Ogasawara, Tetsuhiro Chiba, Yoshihiko Ooka, Naoya Kanogawa, Tenyu Motoyama, Eiichiro Suzuki, Akinobu Tawada, Ryosaku Azemoto, Masami Shinozaki, Masaharu Yoshikawa, Osamu Yokosuka

**Affiliations:** 1 Department of Gastroenterology and Nephrology, Graduate School of Medicine, Chiba University, Chiba, Japan; 2 Department of Gastroenterology, Kimitsu Chuo Hospital, Chiba, Japan; 3 Department of Gastroenterology, Numazu City Hospital, Shizuoka, Japan; National Yang-Ming University, TAIWAN

## Abstract

**Background:**

Intermediate-stage hepatocellular carcinoma (HCC), defined according to the Barcelona Clinic Liver Cancer (BCLC) staging system, is a heterogeneous condition with variable clinical benefits from transarterial chemoembolization (TACE). This study aimed to develop a simple validated prognostic score based on the predictive factors for survival in patients with intermediate-stage HCC treated with TACE.

**Methods:**

Three-hundred and fifty patients with intermediate-stage HCC undergoing initial TACE at Chiba University Hospital (training cohort; *n* = 187) and two affiliated hospitals (validation cohort; *n* = 163) were included. Following variables were entered into univariate and multivariate Cox regression models to develop a points-based clinical scoring system: gender, age, etiology, pretreatment, Child–Pugh score, aspartate aminotransferase, creatinine, C-reactive protein, alfa-fetoprotein, size of the largest lesion, and number and location of lesions.

**Results:**

The number of lesions and the Child–Pugh score were identified as independent prognostic factors in the training cohort. The development of a 0–7-point prognostic score, named the Chiba HCC in intermediate-stage prognostic (CHIP) score, was based on the sum of three subscale scores (Child–Pugh score = 0, 1, 2, or 3, respectively, number of lesions = 0, 2, or 3, respectively, HCV-RNA positivity = 0 or 1, respectively). The generated scores were then differentiated into five groups (0–2 points, 3 points, 4 points, 5 points, and 6–7 points) by the median survival time (65.2, 29.2, 24.3, 13.1, and 8.4 months, respectively; *p* < 0.0001). These results were confirmed in the external validation cohort (*p* < 0.0001).

**Conclusions:**

The CHIP score is easy-to-use and may assist in finding an appropriate treatment strategy for intermediate-stage HCC.

## Introduction

Hepatocellular carcinoma (HCC) is the sixth most common cancer worldwide and the third most common cause of cancer mortality [1]. Transarterial chemoembolization (TACE) is a widely recommended treatment strategy for patients with asymptomatic large or multinodular HCC without macrovascular invasion or extrahepatic metastasis (intermediate-stage HCC) [2–6]. However, because patients with intermediate-stage HCC comprise a heterogeneous population, with differences in tumor size, number, liver function, and possible factors, the clinical benefits of TACE are variable. Therefore, there is a need to construct a treatment strategy for patients with intermediate-stage HCC that is based on prognosis. Furthermore, we are unaware of any prognostic scores based on the statistical analysis of patients with intermediate-stage HCC who received TACE. Using Cox regression analysis, this study aimed to identify the predictive factors for survival in patients with intermediate-stage HCC who received TACE. In addition, we tried to develop a validated prognostic score in those patients.

## Patients and Methods

### Ethics statement

This study was approved by the Research Ethics Committees of Graduate School of Medicine, Chiba University (approval number 1,807), Kimitsu Chuo Hospital (approval number 219) and Numazu City Hospital. Informed consent was not obtained because of the retrospective design. Patient records/information was anonymized and de-identified prior to analysis.

### Patient eligibility

Patients with HCC who underwent initial TACE were retrospectively identified from databases at Chiba University Hospital (between September 2001 and August 2011) and two affiliated hospitals (Kimitsu Chuo Hospital and Numazu City Hospital; between July 2003 and August 2011). We identified all patients histologically or radiologically diagnosed with HCC according to the diagnostic criteria of the American Association for the Study of Liver Diseases [2, 3]. We included patients with intermediate-stage HCC [Barcelona-Clinic Liver Cancer (BCLC) stage B; defined as >3 tumors of any size, 2–3 tumors exceeding a 3–cm diameter, or a single unresectable tumor >5 cm, without macrovascular invasion or extrahepatic spread, and Eastern Cooperative Oncology Group Performance Status = 0]. We excluded patients with HCC at BCLC stages A or C, and patients at BCLC stage B who converted to systemic therapy (including sorafenib and hepatic arterial chemo infusion) during treatment. We identified the predictive factors and developed the prognostic score based on the dataset from Chiba University Hospital (training dataset). The prognostic score was validated using an external and independent cohort from the Kimitsu Chuo Hospital and Numazu City Hospital datasets (validation dataset).

### Indication and strategy for TACE

The treatment policy for patients with intermediate-stage HCC followed our standard practice. Initially, we consider whether definitive treatment can be accomplished with either surgical resection or local ablation. All remaining cases are considered for TACE, the first-line non-curative treatment for intermediate-stage HCC [7]. TACE procedures are performed on demand, with repeated TACE performed if a viable tumor is identified or if there is local or distant intrahepatic recurrence (conventional TACE). For the procedure, a microcatheter is inserted coaxially via a 4–6-French gauge catheter through the femoral artery, and TACE is performed using a super-selective technique [8]. A mixture of ethiodized oil (Lipiodol) and an anticancer agent (epirubicin, cisplatin, or miriplatin) is injected through the tumor-feeding branch. After injection of the Lipiodol and anticancer agent mixture, gelatin sponge particles are injected to obstruct the tumor-feeding branch completely. Because there is a lack of high quality evidence informing selection of TACE agent, the anticancer agent selection strategy at our institution was as follows: (1) epirubicin was selected as first-line treatment before cisplatin approval; (2) cisplatin was selected as first-line treatment between cisplatin approval and miriplatin approval; (3) miripratin was selected as first-line treatment after miripratin approval, and (4) changing anticancer agents was allowed in cases where insufficient therapeutic effect following the previous TACE was demonstrated. A computed tomography scan is performed within 3 months of TACE to evaluate the radiological response of the tumor. Follow-up computed tomography or magnetic resonance imaging is performed every 3–4 months.

### Statistical analysis

Overall survival (OS) was defined as the time from the first TACE to either death from any cause or the date of last follow-up. Univariate and multivariate Cox proportional-hazard regression models were used to estimate the hazard ratios for the risk factors in relation to OS. To develop the prognostic score, we compared models with all possible variable combinations based on the Akaike Information Criterion. The model with the smallest Akaike Information Criterion value was selected as the final model. The prognostic score was then established based on the rounding values of estimated coefficients of the final model. Between-group differences in patient characteristics were analyzed with the Fisher exact test for categorical variables. Survival estimates were derived by the Kaplan–Meier method. All statistical tests were two-sided, and 95% confidence intervals were calculated.

All statistical analyses were performed using SAS software (version 9.3; SAS Institute, Inc., Cary, NC, USA). Proportions for categorical variables were analyzed with the FREQ procedure. The Kaplan–Meier estimates were calculated with the LIFETEST procedure. The Cox proportional-hazards regression models were performed with the PHREG procedure.

### Comparison of different sub-classification models to predict mortality

We compared the developed prognostic score with the following three sub-classification models: the Bolondi model [9], the hepatoma arterial-embolisation prognostic (HAP) score [10] and the Yamakado models [11]. We then compared the concordance index to evaluate the discriminatory ability of the prognostic scores to predict OS [12]. The concordance indexes were calculated with the R survC1 package.

## Results

### Population characteristics

**Fig 1:**
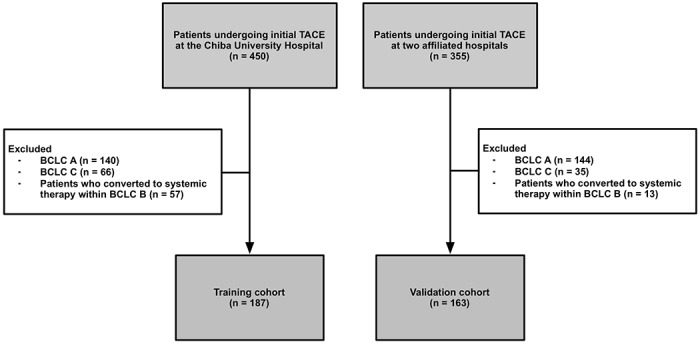
Flow chart of study.

Of the 805 patients with HCC who received TACE in three institutions, we included 350 patients with intermediate-stage HCC undergoing initial TACE for the training cohort (*n* = 187) and the validation cohort (*n* = 163) (Fig 1 and S1 Table). The training cohort had the following characteristics: 74% were men; the median age was 70 years; most patients (70%) had positive serum hepatitis C virus (HCV)-RNA tests; Child–Pugh scores were 5, 6, 7, and 8–9 in 46%, 37%, 13%, and 5% patients, respectively; and 107 patients (57%) received pre-treatment. The validation cohort had the following characteristics: most patients were men (71%); the median age was 71 years; 77% of patients had positive HCV-RNA tests; Child–Pugh scores were 5, 6, 7, and 8–9 in 38%, 31%, 15%, and 15% patients, respectively; and 87 patients (53%) received pre-treatment. The training and validation cohorts were similar with regard to most variables, although the validation cohort had a large number of patients with Child-Pugh scores of 8–9 (Table 1).

**Table 1:** Patient characteristics.

	Training Cohort	Validation Cohort	*P*
**Number of patients**	187	163	
**Gender** [*n* (%)]			
Male	139 (74)	115 (71)	0.472
Female	48 (26)	48 (29)	
**Age, years** [*n* (%)]			
≤70	85 (46)	69 (36)	0.065
>70	102 (54)	94 (64)	
Median (range)	70 (30–88)	71 (52–89)	
**Child–Pugh score** [*n* (%)]			
5	85 (46)	62 (38)	0.006
6	69 (37)	51 (31)	
7	24 (13)	25 (15)	
8–9	9 (5)	25 (15)	
**HBV-DNA positive** [*n* (%)]			
Absent	166 (89)	154 (94)	0.084
Present	21 (11)	9 (6)	
**HCV-RNA positive** [*n* (%)]			
Absent	56 (30)	37 (23)	0.117
Present	131 (70)	126 (77)	
**Alcohol > 80 g/day** [*n* (%)]			
Absent	150 (80)	138 (85)	0.326
Present	37 (20)	25 (15)	
**Maximum tumor size, mm** [*n* (%)]			
≤30	81 (43)	74 (45)	0.288
>30, ≤50	72 (39)	51 (31)	
>50	34 (18)	38 (23)	
Median (range)	32 (10–147)	32 (10–150)	
**Number of liver tumors** [*n* (%)]			
1	16 (9)	12 (7)	0.636
2–7	140 (75)	118 (72)	
≥8	31 (17)	33 (20)	
**Location of liver tumors** [*n* (%)]			
Uni-lobar	89 (48)	70 (43)	0.392
Bi-lobar	98 (52)	93 (57)	
**AFP, ng/mL** [*n* (%)]			
≤400	141 (75)	126 (77)	0.707
>400	46 (25)	37 (23)	
Median (range)	43.7 (1.1–112,300)	43.5 (0.6–113,000)	
**Pre-treatment** [*n* (%)]			
Absent	80 (43)	76 (47)	0.518
Present	107 (57)	87 (53)	

Abbreviations: AFP, alpha-fetoprotein.

### Univariate and multivariate survival analyses

During the study period, 117 of 187 patients in the training cohort and 136 of 163 patients in validation cohort died. The median OSs were 27.8 (95% CI: 23.5–32.2) and 20.5 (95% CI: 17.9–23.1) months for the training and validation cohorts, respectively. The median follow-up periods were 22.8 and 19.2 months for the training and validation cohorts, respectively. No significant differences were observed in overall survival (OS) regardless of timing of initial TACE (S2 Table).

Next, log-relative risk of death related to the size of the largest lesion and the number of lesions was examined (S1 Fig). We divided the size of the largest lesion into ≤30 mm, >30 mm and ≤50 mm, >50 mm (multi-nodule), and >50 mm (single nodule) categories; the data suggests that patients with a single nodule were at low risk of death. However, there was no clear discrepancy for log-relative risk between the sizes of the tumors in patients with multi-nodule intermediate-stage HCC. Consequently, we defined 50 mm as the cut-off value for the size of the largest lesion in univariate analysis. We then divided the number of lesions into 4 categories (1, 2–4, 5–7, and ≥8 lesions). This revealed clear discrepancies in log-relative risks between the patients with single lesion, 2–7 lesions, and ≥8 lesions.

In univariate analysis, HCV-RNA positivity, Child–Pugh score, maximum tumor size, number of liver tumors, location of liver tumors, and pre-treatment were identified as statistically significant predictors (Table 2). We subsequently identified independent variables by comparing all possible combination of variables based on the AIC. Smallest AIC values were selected for the final model. In the multivariate survival analysis, HCV-RNA positivity, Child–Pugh score and number of liver tumors remained significant predictive factors.

**Table 2:** Univariate and multivariate survival analyses for clinical variables.

	Univariate analysis				Multivariate		
Variables	*n*	Hazard ratio	95% C.I.	*P* value	Hazard ratio	95% C.I.	*P* value
**Gender**							
Male	139	Reference					
Female	48	0.798	0.511–1.244	0.319			
**Age, years**							
≤70	85	Reference					
>70	102	1.184	0.820–1.709	0.368			
**HBV-DNA positive**							
Absent	166	Reference					
Present	21	0.663	0.356–1.236	0.196			
**HCV-RNA positive**							
Absent	56	Reference			Reference		
Present	131	1.773	1.158–2.715	0.008	1.971	1.241–3.132	0.004
**Alcohol abuse**							
Absent	150	Reference					
Present	37	1.012	0.641–1.598	0.960			
**Child–Pugh score**							
5	85	Reference			Reference		
6	64	1.665	1.096–2.530	0.017	1.572	1.026–2.410	0.038
7	24	3.828	2.146–6.827	< 0.001	3.850	2.057–7.205	< 0.001
8–9	9	8.420	3.922–18.077	< 0.01	8.089	3.452–18.955	< 0.001
**Creatinine**							
≤ UNL	162	Reference					
> UNL	25	1.513	0.877–2.611	0.137			
**AST**							
≤ UNL	34	Reference					
> UNL	153	1.491	0.920–2.419	0.105			
**CRP**							
≤ UNL	139	Reference					
> UNL	48	1.257	0.831–1.902	0.279			
**AFP, ng/mL**							
≤400	141	Reference					
>400	46	1.063	0.698–1.620	0.776			
**Maximum tumor size, mm**							
≤50	153	Reference					
>50	34	0.535	0.315–0.907	0.020			
**Number of liver tumors**							
1	16	Reference			Reference		
2–7	140	4.115	1.508–11.230	0.006	4.770	1.736–13.110	0.003
≥8	31	9.194	3.153–26.810	< 0.001	6.895	2.311–20.570	0.001
**Location of liver tumors**							
Uni-lobar	89	Reference					
Bi-lobar	98	1.676	1.157–2.428	0.006			
**Pre-treatment**							
Absent	80	Reference					
Present	107	1.994	1.361–2.923	< 0.001			

Abbreviations: UNL, upper normal limit; AST, Aspartate aminotransferase; CRP, C-reactive protein; AFP, alpha-fetoprotein.

### The prognostic score as a predictor of OS

**Fig 2:**
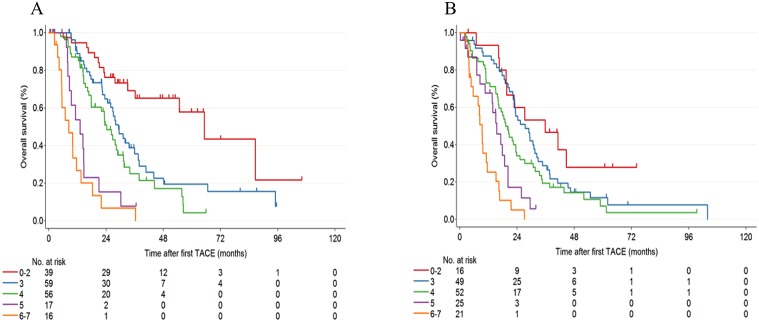
Kaplan–Meier survival curves according to the CHIP score in the training dataset (A) and the validation dataset (B).

The prognostic score was developed based on the rounding values of estimated coefficients of the final model. As a result, the three predictive factors of OS in the multivariate survival analysis, namely HCV-RNA positivity, Child–Pugh score and number of lesions, were used for the score calculation (Table 3). To generate a simple and easy-to-use score model, we made the “additive” formula using logarithmically-transformed hazard ratios. Scores were calculated on the basis of the formula as follows: score = round [1.5 × ln(HR)] (S3 Table). The final score, named the Chiba HCC in intermediate-stage prognostic (CHIP) score, was defined as the sum of the three sub-scores with a minimum score of 0 and a maximum score of 7. Among the training cohort, 39, 59, 56, 17, and 16 patients showed scores of 0–2, 3, 4, 5, and 6–7, respectively. Similarly, among the validation cohort, 16, 49, 52, 25, and 21 patients showed scores of 0–2, 3, 4, 5, and 6–7, respectively. The observed cumulative survival of patients grouped by score was calculated using the Kaplan–Meier method for both groups (Fig 2). The CHIP score successfully identified five subgroups with distinct prognoses (training cohort: *p* < 0.0001, validation cohort: *p* < 0.0001).

**Table 3:** Calculation of the prognostic score in patients with intermediate-stage hepatocellular carcinoma.

Prognostic factor	Points
**Child-Pugh score**	
5	0
6	1
7	2
8–9	3
**Number of liver tumors**	
1	0
2–7	2
≥8	3
**HCV-RNA positive**	
Absent	0
Present	1

Abbreviation: HCV, hepatitis C virus

### Comparison of different sub-classification models to predict mortality

**Fig 3:**
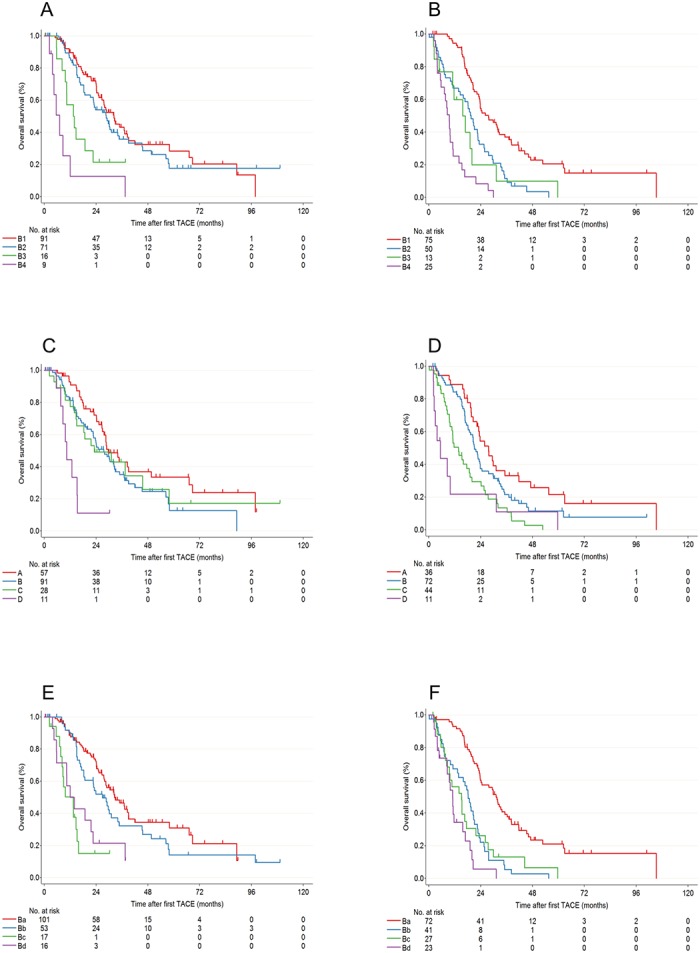
Kaplan-Meier survival curves according to the Bolondi model (A: training dataset, B: validation dataset), the HAP score (C: training dataset, D: validation dataset), and the Yamakado model (E: training dataset, F: validation dataset).

Kaplan–Meier curves was also analyzed according to the Bolondi model, the HAP score and the Yamakado method (Fig 3). All of these sub-classification models were found to be significant in the log-rank test using the training and validation datasets (S4 Table). The discriminatory value of the various prognostic scores to predict mortality was evaluated separately using both the training and validation datasets by the concordance index. In the training dataset, the concordance index of CHIP score was the highest and significantly better than the Bolondi model and the Yamakado model (Table 4). However, there were no significant differences between CHIP score and the other three scoring models in the validation dataset (Table 5).

**Table 4:** Concordance index (C-index) for each scoring model in the training set.

Scoring system	C-index	95% C.I.	Difference between our prognosis scoring system	95% C.I.	P-value
**CHIP score**	0.686	0.607–0.764	0.000	-	-
**Bolondi model**	0.575	0.490–0.659	-0.111	-0.210 –-0.012	0.028
**HAP score**	0.586	0.453–0.718	-0.100	-0.211 –-0.010	0.075
**Yamakado model**	0.611	0.546–0.676	-0.075	-0.140 –-0.010	0.023

Abbreviations: CHIP score, Chiba hepatocellular carcinoma in intermediate-stage prognostic score; HAP score, hepatoma arterial-embolisation prognostic score.

**Table 5:** Concordance index (C-index) for each scoring model in the validation set.

Scoring system	C-index	95% C.I.	Difference between our prognosis scoring system	95% C.I.	P-value
**CHIP score**	0.655	0.607–0.703	0.000	-	-
**Bolondi model**	0.628	0.568–0.688	-0.026	-0.099–0.046	0.476
**HAP score**	0.665	0.624–0.707	0.011	-0.040–0.061	0.677
**Yamakado model**	0.669	0.626–0.712	0.014	–0.036–0.064	0.576

Abbreviations: CHIP score, Chiba hepatocellular carcinoma in intermediate-stage prognostic score; HAP score, hepatoma arterial-embolisation prognostic score.

## Discussion

This study aimed to establish a simple scoring model that could predict the prognosis of patients with intermediate-stage HCC receiving TACE. The multivariate analysis successfully showed that the HCV RNA positivity, the Child–Pugh score and the number of tumors were independently and significantly associated with the prognosis of intermediate-stage HCC treated with TACE. Although no HCV patients received antiviral treatment, 12 of 21 HBV patients in the training set received nucleos(t)ide analogues. Considering only a small proportion of patients in this study received antiviral therapy, it is difficult to determine the utility of our prognostic score regarding previous antiviral therapy. Further analyses in a larger number of patients would be needed to clarify this issue.

Subsequently, we divided the Child–Pugh score, an internationally recognized index of liver function, into four categories in the CHIP score (5, 6, 7, and 8–9). Additionally, we found that the number of tumors was best divided into three categories (1, 2–7, and ≥8 tumors), which is consistent with current knowledge. For example, the number of tumors is known to be associated with intrahepatic spread of malignant cells, and is consistently shown to influence survival [13,14]. Additionally, patients with a single unresectable nodule have good prognoses regardless of whether they receive TACE [15,16]. We made single tumor an independent category because our data also suggested that patients with such tumors had good prognoses. Furthermore, when comparing patients with ≥8 tumors and those with 2–7 tumors, we found that prognoses were poorer in the former. As 140 patients in the training set (75%) had between 2 and 7 lesions, we attempted to further stratify these patients. We performed univariate survival analysis for overall survival according to number of lesions (2–4 tumors vs. 5–7 tumors). However, the hazard ratio in patients with 2–4 tumors was almost identical to that in patients with 5–7 patients (4.182 vs. 3.969). Further analyses would be necessary to examine whether HCC patients with 2–7 tumors could be divided into subgroups.

Bolondi et al. divided the BCLC stage B into B1–B4 sub-classifications based on existing reports, trials, and expert opinion [9]. Their method was validated in several reports in BCLC stage B patients who received TACE [17, 18]. Importantly, tumor burden according to the Bolondi model is determined by the up-to–7 criteria, sum of the size of the largest tumor (in cm) and the number of tumors. In contrast, CHIP score takes into account tumor number, but not tumor size. For example, a HCV-negative Child–Pugh A patient complicated with a single HCC with a diameter of 10 cm was classified into the group with most favorable prognosis according to our score system (score 0) but not according to the Bolondi model (class B2). Moreover, the up-to-7 criteria itself was established based on the large amount of liver transplantation data performed without the Milan criteria [19]. Although Bolondi’s sub-classification method is not specific prognostication in BCLC stage B patients who received TACE, it does allow sub-division of treatment options and better prediction of the associated patient outcomes. The HAP score is a simple scoring index requiring the measurement of two tumor variables (alpha-fetoprotein and the largest size of tumor) and two liver variables (albumin and bilirubin) and can predict outcomes in transcatheter arterial embolization/TACE [10]. However, their analysis comprised both patients with BCLC stage B and those with BCLC stage A (35%), BCLC stage C, (31%), and BCLC stage D (4%), which would influence the usefulness of the final model. Yamakado et al. also analyzed patients with BCLC stage B who received TACE, but divided them into stages Ba–Bd [11]. Their method created four groups according to the Child–Pugh class and a combination of tumor size and number. Although that model could predict the prognosis of patients with BCLC stage B that received TACE, it was not based on the Cox regression model.

The CHIP score is specific for patients with intermediate-stage HCC who receive TACE and that is based on a Cox regression model and established the rounding value of estimated coefficients of the final model. This appears to predict survival in patients with intermediate-stage HCC who receive TACE at least equally to the Bolondi model, the HAP score and the Yamakado model. Indeed, the concordance index in both the training and validation datasets was highest for the CHIP score. Furthermore, this new prognostic score could sub-categorize patients with intermediate-stage HCC into five categories, which is greater than the number categories in existing methods. The prognostic value for our datasets was comparable to existing criteria. However, the CHIP score may also be able to predict survival. Recently, Sieghart et al. described a meaningful scoring system, designated the ART score. The score comprises increase in Child-Pugh score, increase in AST, and radiological response to first TACE in patients with HCC. This scoring system may assist in decision making regarding TACE retreatment by estimating prognosis following second TACE [20]. Unlike the CHIP score, the purpose of the ART score was not subclassification of intermediate stage HCC.

The techniques and procedures used to deliver TACE are highly variable between institutions [21]. The CHIP score was equally discriminatory in the validation dataset despite differences in technique and patient characteristics. However, in both the training and validation datasets, all cases employed conventional TACE with a super-selective technique. Although most TACE in Asia (particularly Japan) is performed using this technique, it is not the main practice in Western countries where drug-eluting bead TACE is widely used. The CHIP score should therefore be validated against another external dataset treated with drug-eluting bead TACE.

In summary, we developed a novel prognostic score specifically for patients with intermediate-stage HCC undergoing TACE. The prognostic score is simple to employ and is based on just two variables, the Child–Pugh score and the number of tumors. Although validated in an independent dataset, a large prospective cohort will be necessary to confirm our results.

## Supporting Information

S1 Fig Natural logarithm of relative risk for death related to the size of the largest tumor (A; reference, ≤30 mm) and the number of tumors (B; reference, single nodule). (TIF)

S1 Table The clinical data of the study participants. (XLSX)

S2 Table Univariate analysis for the overall survival with Cox proportional hazards model in view of the timing of initial TACE. (DOC)

S3 Table Development of the CHIP score. (DOC)

S4 Table Median survival time for each scoring model. (DOC)

## References

[1] Ferlay J, Shin HR, Bray F, Forman D, Mathers C, Parkin DM (2010) Estimates of worldwide burden of cancer in 2008: GLOBOCAN 2008. Int J Cancer 127: 2893–917. 10.1002/ijc.25516 21351269

[2] Bruix J, Sherman M (2005) Practice Guidelines Committee, American Association for the Study of Liver Diseases. Management of hepatocellular carcinoma. Hepatology 42: 1208–36. 16250051 10.1002/hep.20933

[3] Bruix J, Sherman M (2011) American Association for the Study of Liver Diseases. Management of hepatocellular carcinoma: an update. Hepatology 53: 1020–2. 10.1002/hep.24199 21374666 PMC3084991

[4] European Association For The Study Of The Liver; European Organisation For Research And Treatment Of Cancer (2012) EASL-EORTC clinical practice guidelines: management of hepatocellular carcinoma. J Hepatol 56: 908–43. 10.1016/j.jhep.2011.12.001 22424438

[5] Kudo M, Izumi N, Kokudo N, Matsui O, Sakamoto M, Nakashima O, et al (2011) Management of hepatocellular carcinoma in Japan: Consensus-Based Clinical Practice Guidelines proposed by the Japan Society of Hepatology (JSH) 2010 updated version. Dig Dis 29: 339–64. 10.1159/000327577 21829027

[6] Omata M, Lesmana LA, Tateishi R, Chen PJ, Lin SM, Yoshida H, et al (2011) Asian Pacific Association for the Study of the Liver consensus recommendations on hepatocellular carcinoma. Hepatol Int 4: 439–74. 10.1007/s12072-010-9165-7 PMC2900561 20827404

[7] Golfieri R, Cappelli A, Cucchetti A, Piscaglia F, Carpenzano M, Peri E, et al (2011) Efficacy of selective transarterial chemoembolization in inducing tumor necrosis in small (<5 cm) hepatocellular carcinomas. Hepatology 53: 1580–9. 10.1002/hep.24246 21351114

[8] Takayasu K, Arii S, Kudo M, Ichida T, Matsui O, Izumi N, et al (2012) Superselective transarterial chemoembolization for hepatocellular carcinoma. Validation of treatment algorithm proposed by Japanese guidelines. J Hepatol 56: 886–92. 10.1016/j.jhep.2011.10.021 22173160

[9] Bolondi L, Burroughs A, Dufour JF, Galle PR, Mazzaferro V, Piscaglia F, et al (2012) Heterogeneity of patients with intermediate (BCLC B) Hepatocellular Carcinoma: proposal for a subclassification to facilitate treatment decisions. Semin Liver Dis 32: 348–59. 10.1055/s-0032-1329906 23397536

[10] Kadalayil L, Benini R, Pallan L, O'Beirne J, Marelli L, Yu D, et al (2013) A simple prognostic scoring system for patients receiving transarterial embolisation for hepatocellular cancer. Ann Oncol 24: 2565–70. 10.1093/annonc/mdt247 23857958 PMC4023407

[11] Yamakado K, Miyayama S, Hirota S, Mizunuma K, Nakamura K, Inaba Y, et al (2014) Subgrouping of intermediate-stage (BCLC stage B) hepatocellular carcinoma based on tumor number and size and Child–Pugh grade correlated with prognosis after transarterial chemoembolization. Jpn J Radiol 32: 260–5. 10.1007/s11604-014-0298-9 24615165

[12] Uno H, Cai T, Pencina MJ, D'Agostino RB, Wei LJ (2011) On the C-statistics for evaluating overall adequacy of risk prediction procedures with censored survival data. Stat Med 30: 1105–17. 10.1002/sim.4154 21484848 PMC3079915

[13] Poon RT, Fan ST, Ng IO, Wong J (2003) Prognosis after hepatic resection for stage IVA hepatocellular carcinoma: a need for reclassification. Ann Surg 237: 376–83. 12616122 10.1097/01.SLA.0000055224.68432.80 PMC1514304

[14] Gao HJ, Zhang YJ, Chen MS, Chen MX, Huang JT, Xu Li, et al (2014) Rationality and effectiveness of transarterial chemoembolization as an initial treatment for BCLC B stage HBV-related hepatocellular carcinoma. Liver Int 34: 612–20. 10.1111/liv.12307 24028297

[15] Dumortier J, Chapuis F, Borson O, Davril B, Scoazec JY, Poncet G, et al (2006) Unresectable hepatocellular carcinoma: survival and prognostic factors after lipiodol chemoembolisation in 89 patients. Dig Liver Dis 38: 125–33. 16389002 10.1016/j.dld.2005.10.025

[16] Xiao J, Li G, Lin S, He K, Lai H, Mo X, et al (2014) Prognostic factors of hepatocellular carcinoma patients treated by transarterial chemoembolization. Int J Clin Exp Pathol 7: 1114–23. 24696728 PMC3971316

[17] Ha Y, Shim JH, Kim SO, Kim KM, Lim YS, Lee HC (2014) Clinical appraisal of the recently proposed Barcelona Clinic Liver Cancer stage B subclassification by survival analysis. J Gastroenterol Hepatol 29: 787–93. 10.1111/jgh.12452 24224567

[18] Wang JH, Kee KM, Lin CY, Hung CH, Chen CH, Lee CM, et al (2014) Validation and modification of a proposed sub-staging system for patients with intermediate hepatocellular carcinoma. J Gastroenterol Hepatol 30: 358–63. 10.1111/jgh.12686 25088668

[19] Mazzaferro V, Llovet JM, Miceli R, Bhoori S, Schiavo M, Mariani L, et al (2009) Predicting survival after liver transplantation in patients with hepatocellular carcinoma beyond the Milan criteria: a retrospective, exploratory analysis. Lancet Oncol 10: 35–43. 10.1016/S1470-2045(08)70284-5 19058754

[20] Sieghart W, Hucke F, Pinter M, Graziadei I, Vogel W, Müller C, et al (2013) The ART of decision making: retreatment with transarterial chemoembolization in patients with hepatocellular carcinoma. Hepatology 57: 2261–73. 10.1002/hep.26256 23316013

[21] Marelli L, Stigliano R, Triantos C, Senzolo M, Cholongitas E, Davies N, et al (2007) Transarterial therapy for hepatocellular carcinoma: which technique is more effective? A systematic review of cohort and randomized studies. Cardiovasc Intervent Radiol 30: 6–25. 17103105 10.1007/s00270-006-0062-3

