# Supplementary figures and images for: A Prognostic Score for Patients with Intermediate-Stage Hepatocellular Carcinoma Treated with Transarterial Chemoembolization
Source: PLoS One. 2015 Apr 28;10(4):e0125244. doi: 10.1371/journal.pone.0125244 (PMC4412579; doi:10.1371/journal.pone.0125244)

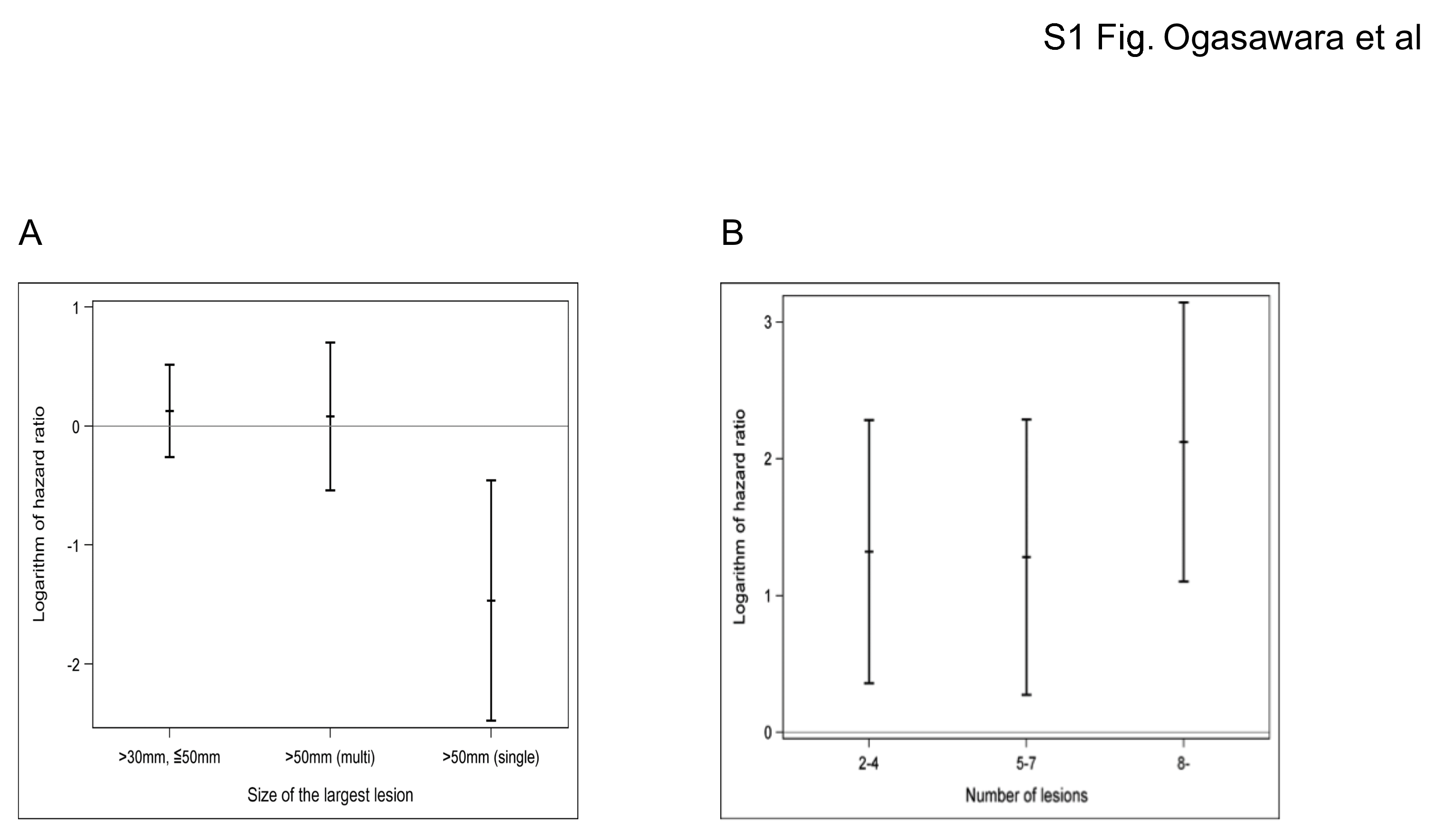

Supplement: S1 Fig — (TIF) [file pone.0125244.s001.tif]
