# Supplementary material for: A Prognostic Score for Patients with Intermediate-Stage Hepatocellular Carcinoma Treated with Transarterial Chemoembolization
Source: PLoS One. 2015 Apr 28;10(4):e0125244. doi: 10.1371/journal.pone.0125244 (PMC4412579; doi:10.1371/journal.pone.0125244)
Supplement: S2 Table — (DOC) [file pone.0125244.s003.doc]

**S2 Table**. Univariate analysis for the overall survival with Cox proportional hazards model in view of the timing of initial TACE

A. Training dataset (*n* = 187)

| **Variables** | **Univariate analysis (*n* = 187)** | | ***P* value** |
| --- | --- | --- | --- |
|  | **Hazard ratio** | **95% C.I.** |  |
| **Year** |  |  |  |
| 2001–2003 | Reference |  |  |
| 2004–2005 | 0.836 | 0.424–1.608 | 0.573 |
| 2006–2007 | 1.132 | 0.619–2.071 | 0.687 |
| 2008–2009 | 1.037 | 0.589–1.826 | 0.901 |
| 2010–2011 | 0.915 | 0.472–1.771 | 0.791 |

B. Validation dataset (*n* = 163)

| **Variables** | **Univariate analysis (*n* = 163)** | | ***P* value** |
| --- | --- | --- | --- |
|  | **Hazard ratio** | **95% C.I.** |  |
| **Year** |  |  |  |
| 2003 | Reference |  |  |
| 2004–2005 | 1.140 | 0.498–2.613 | 0.756 |
| 2006–2007 | 0.986 | 0.439–2.213 | 0.973 |
| 2008–2009 | 0.940 | 0.418­–2.115 | 0.881 |
| 2010–2011 | 0.879 | 0.372–2.078 | 0.769 |
