# Supplementary material for: A Prognostic Score for Patients with Intermediate-Stage Hepatocellular Carcinoma Treated with Transarterial Chemoembolization
Source: PLoS One. 2015 Apr 28;10(4):e0125244. doi: 10.1371/journal.pone.0125244 (PMC4412579; doi:10.1371/journal.pone.0125244)
Supplement: S3 Table — (DOC) [file pone.0125244.s004.doc]

**S3 Table.** Computing process of the CHIP score

| **Variable** | **Hazard ratio** | **In (HR) =**  **estimated coefficient** | **1.5 x In(HR)** | **Rounding**  **(score)** |
| --- | --- | --- | --- | --- |
| **HCV-RNA positive** |  |  |  |  |
| Absent | 1.000 | 0.000 | 0.000 | 0.000 |
| Present | 1.949 | 0.667 | 1.001 | 1.000 |
| **Child–Pugh score** |  |  |  |  |
| 5 | 1.000 | 0.000 | 0.000 | 0.000 |
| 6 | 1.569 | 0.450 | 0.676 | 1.000 |
| 7 | 3.897 | 1.360 | 2.040 | 2.000 |
| ≥8 | 8.374 | 2.125 | 3.188 | 3.000 |
| **Number of liver tumors** |  |  |  |  |
| 1 | 1.000 | 0.000 | 0.000 | 0.000 |
| 2-7 | 4.258 | 1.449 | 2.173 | 2.000 |
| ≥8 | 6.228 | 1.829 | 2.744 | 3.000 |

Formula of the score = round [1.5 x ln(HR)]

Abbreviations: CHIP score, Chiba hepatocellular carcinoma in intermediate-stage prognostic score; HCV, hepatitis C virus; HR, hazard ratio.
