# Supplementary material for: A Prognostic Score for Patients with Intermediate-Stage Hepatocellular Carcinoma Treated with Transarterial Chemoembolization
Source: PLoS One. 2015 Apr 28;10(4):e0125244. doi: 10.1371/journal.pone.0125244 (PMC4412579; doi:10.1371/journal.pone.0125244)
Supplement: S4 Table — (DOC) [file pone.0125244.s005.doc]

**S4 Table**. Median survival time for each scoring model

A. CHIP score

|  | **Training dataset (n = 187)** | | **Validation dataset (n = 163)** | |
| --- | --- | --- | --- | --- |
| **Score** | **Overall**  **survival** | **95% C.I.** | **Overall**  **survival** | **95% C.I.** |
| **0–2** | 65.2 | 36.2 – undefined | 35.7 | 16.5 – undefined |
| **3** | 29.2 | 24.6 – 35.9 | 27.4 | 22.3 – 31.4 |
| **4** | 24.3 | 17.7 – 28.8 | 19.7 | 16.1 – 23.5 |
| **5** | 13.1 | 8.0 – 14.7 | 15.5 | 8.5 – 18.8 |
| **6–7** | 8.4 | 5.3 – 11.6 | 9.4 | 4.7 – 11.5 |
| **P-value**  **(Log-rank)** | < 0.0001 |  | < 0.0001 |  |

B. HAP score

|  | **Training dataset (n = 187)** | | **Validation dataset (n = 163)** | |
| --- | --- | --- | --- | --- |
| **Class** | **Overall**  **survival** | **95% C.I.** | **Overall**  **survival** | **95% C.I.** |
| **A** | 29.7 | 26.7 – 49.6 | 27.7 | 20.5 – 35.2 |
| **B** | 27.6 | 22.6 – 32.5 | 21.1 | 18.2 – 23.8 |
| **C** | 23.2 | 14.7 – 45.5 | 13.7 | 9.6 – 18.7 |
| **D** | 10.4 | 5.5 – 15.3 | 5.4 | 2.2 – 31.2 |
| **P-value**  **(Log-rank)** | 0.0002 |  | < 0.0001 |  |

C. Bolondi model

|  | **Training dataset (n = 187)** | | **Validation dataset (n = 163)** | |
| --- | --- | --- | --- | --- |
| **Class** | **Overall**  **survival** | **95% C.I.** | **Overall**  **survival** | **95% C.I.** |
| **B1** | 32.3 | 25.3 – 38.5 | 27.7 | 23.3 – 32.7 |
| **B2** | 28.6 | 21.3 – 34.0 | 19.5 | 13.7 – 22.7 |
| **B3** | 13.7 | 8.2 – 22.6 | 15.7 | 4.8 – 19.9 |
| **B4** | 7.1 | 2.4 – 11.9 | 9.5 | 5.4 – 11.1 |
| **P-value**  **(Log-rank)** | < 0.0001 |  | < 0.0001 |  |

D. Yamakado model

|  | **Training dataset (n = 187)** | | **Validation dataset (n = 163)** | |
| --- | --- | --- | --- | --- |
| **Class** | **Overall**  **survival** | **95% C.I.** | **Overall**  **survival** | **95% C.I.** |
| **Ba** | 32.5 | 27.8 – 39.0 | 31.1 | 23.7 – 36.3 |
| **Bb** | 26.9 | 18.3 – 34.0 | 18.2 | 13.3 – 21.1 |
| **Bc** | 13.4 | 7.6 – 15.1 | 15.2 | 8.6 – 17.4 |
| **Bd** | 12.9 | 5.6 – 22.6 | 11.0 | 8.3 – 15.7 |
| **P-value**  **(Log-rank)** | < 0.0001 |  | < 0.0001 |  |

Abbreviations: CHIP score, Chiba hepatocellular carcinoma in intermediate-stage prognostic score; HAP score, hepatoma arterial-embolisation prognostic score.
